# Biocompatibility of Bone Marrow-Derived Mesenchymal Stem Cells in the Rat Inner Ear following Trans-Tympanic Administration

**DOI:** 10.3390/jcm9061711

**Published:** 2020-06-02

**Authors:** Adrien A. Eshraghi, Emre Ocak, Angela Zhu, Jeenu Mittal, Camron Davies, David Shahal, Erdogan Bulut, Rahul Sinha, Viraj Shah, Mario M. Perdomo, Rahul Mittal

**Affiliations:** 1 Department of Otolaryngology, Hearing Research Laboratory, University of Miami Miller School of Medicine, Miami, FL 33136, USA; dremreocak@gmail.com (E.O.); zhua@med.miami.edu (A.Z.); j.mittal@med.miami.edu (J.M.); cdavi191@med.fiu.edu (C.D.); dxs1298@miami.edu (D.S.); erdoganbulut@gmail.com (E.B.); rahulsinha3@yahoo.com (R.S.); viraj.shah@med.miami.edu (V.S.); mmp78@miami.edu (M.M.P.); r.mittal11@med.miami.edu (R.M.); 2 Department of Neurological Surgery, University of Miami Miller School of Medicine, Miami, FL 33136, USA; 3 Department of Biomedical Engineering, University of Miami, Coral Gables, FL 33146, USA

**Keywords:** mesenchymal stem cells, inner ear, oxidative stress, caspase 3, proinflammatory cytokines, TUNEL staining

## Abstract

Recent advancements in stem cell therapy have led to an increased interest within the auditory community in exploring the potential of mesenchymal stem cells (MSCs) in the treatment of inner ear disorders. However, the biocompatibility of MSCs with the inner ear, especially when delivered non-surgically and in the immunocompetent cochlea, is not completely understood. In this study, we determined the effect of intratympanic administration of rodent bone marrow MSCs (BM-MSCs) on the inner ear in an immunocompetent rat model. The administration of MSCs did not lead to the generation of any oxidative stress in the rat inner ear. There was no significant production of proinflammatory cytokines, tumor necrosis factor (TNF)-α, interleukin (IL)-1β, IL-6 and IL-12, due to BM-MSCs administration into the rat cochlea. BM-MSCs do not activate caspase 3 pathway, which plays a central role in sensory cell damage. Additionally, transferase dUTP nick end labeling (TUNEL) staining determined that there was no significant cell death associated with the administration of BM-MSCs. The results of the present study suggest that trans-tympanic administration of BM-MSCs does not result in oxidative stress or inflammatory response in the immunocompetent rat cochlea.

## 1. Introduction

Auditory disorders and hearing loss affect a significant proportion of the human population [1,2,3]. Of American adults over age 18, 15% report having some difficulty in hearing [4]. Factors such as age, noise trauma, infection, and ototoxic drugs can lead to damage of sensory auditory hair cells in the inner ear (cochlea), leading to hearing impairment [4,5,6]. Any perturbation of the sound pathway can lead to hearing impairment. However, sensorineural hearing loss (SNHL) predominantly results from damage to the auditory hair cells. The prevalence of SNHL increases with age and it is the most common type of hearing loss in adults over 65 years old, occurring in 23% of adults in that age range [7,8]. Clinically, there is an insensitivity to weak sounds and difficulty understanding speech. Treatment for SNHL depends on the etiology, but there is no universal treatment to prevent or reverse the effects of sensorineural deafness. Management focuses on amplifying remaining auditory function through the use of assistive listening devices such as hearing aids or cochlear implants and avoiding risk factors for further hearing loss, with the goal of improving daily function and quality of life of hearing-impaired individuals [9,10,11]. However, hearing aids are unpopular due to poor fitting, cosmetic issues, and fear of social stigmatization [12,13].

Due to the limitations of external devices to treat inner ear disorders, there has been an increased interest in restoring hearing through stem cells [14,15,16]. In recent years, stem cell therapy is emerging as a promising treatment modality for various auditory disorders [17,18]. Although the potential of induced pluripotent stem cells (iPSCs) has been harnessed in different studies [19,20,21], the biocompatibility of mesenchymal stem cells (MSCs) in the inner ear is not well studied. Stem cells have been studied in other systems as a replacement for damaged cells. MSCs are multipotent cells that are isolated from adipose tissue and bone marrow known to stimulate cell differentiation through secreting mitogenic, angiogenic, anti-apoptotic, and immunomodulatory factors [22]. An advantage to MSCs are their low immunogenicity—undifferentiated MSCs express low levels of human leukocyte antigen (HLA) class I and II molecules, evading recognition by the immune system [23,24]. Additionally, MSCs can be autologously harvested, which mitigates the issue of immunologic rejection by the host. Experimentally, MSCs have been used as a cell source to replenish damaged tissue since they are capable of differentiating into various tissue types in response to environmental cues, such as secreted and insoluble growth factors – tumor growth factor (TGF)-alpha, TGF-beta, fibroblast growth factor (FGF)-2, insulin-like growth factor (IGF)-1, vascular endothelial growth factor (VEGF), epidermal growth factor (EGF) – and chemokines (interleukin (IL)-1, IL2, IL-12, tumor necrosis factor (TNF)-alpha, interferon (IFN)-gamma) [25,26,27]. MSCs are also known to reverse apoptosis in other cell types, such as cardiac myoblasts, neurons, and lung fibroblasts [28,29,30].

Despite their promise, there is a lack of understanding regarding the biocompatibility of MSCs with the inner ear. Most of the previous studies have used complex surgical techniques for delivery of stem cells into the inner ear and under immunosuppression. The objective of this study was to determine whether non-surgical trans-tympanic administration of bone marrow mesenchymal stem cells bone marrow mesenchymal stem cells (BM-MSCs) does not induce oxidative stress as determined by 8-isoprostane immunostaining. We also determined whether BM-MSCs trigger inflammation in terms of proinflammatory cytokine production as well as activating the apoptotic pathway or inducing cell death determined by cleaved caspase 3 and terminal deoxynucleotidyl transferase dUTP nick end labeling (TUNEL) immunostaining, respectively, in the immunocompetent cochlea.

## 2. Materials and Methods

### 2.1. Animals

Both male and female Sprague-Dawley rats (Charles River) weighing between 200–300 g were used in the experiments. The animals were fed on a standard diet and kept on a 12 h light/dark cycle. The animal protocol was approved by the Institutional Animal Care and Use Committee (IACUC) of the University of Miami (Protocol number 17-208; Date of approval 2 November 2017) and is in full compliance with published National Institute of Health (NIH) guidelines for the care and use of laboratory animals.

### 2.2. Experimental Design

The animals were divided into three groups: Group 1 was the control group receiving no experimental treatment. Group 2 was the sham group, receiving 50 µL trans-tympanic administration of phosphate buffered saline (PBS). Group 3 was the experimental group, receiving trans-tympanic administration of 1x 10^5^ BM-MSCs in a total volume of 50 µL of PBS. For trans-tympanic administration, a bolus of BM-MSCs or PBS was slowly injected at pars tensa manually under stero-microscope as described in previous studies [31,32,33,34]. After the injection, the animals were kept in the lateral position with the injected ear oriented upward for 15 min before returning them into cages. Each group had ten rats. Contralateral ear from both groups also served as control. One of the objectives of the study was to determine whether non-invasive administration of MSCs did not induce oxidative stress and inflammation in the inner ear. Therefore, we have not included any groups with intracochlear injection of BM-MSCs which is considered invasive and may have implications in hearing impairment.

### 2.3. Generation of Rat BM-MSCs

Rat BM-MSCs were generated and characterized as described in detail in a previous study [35]. There was an abundant expression of CD90 and absence of CD45 on BM-MSCs determined by flow cytometry after staining with appropriate antibodies confirming their MSC phenotype [36].

### 2.4. 8-Isoprostane Determination

The quantitative levels of 8-isoprostane and cleaved caspase 3 in whole cochlear homogenates were determined by ELISA using commercially available kit as per manufacturer’s instructions (Abcam, Cambridge, MA, USA). The cochlear tissues treated with tert-butyl-hydroperoxide (100 µM) (Sigma Aldrich, St Louis, MO, USA) for 30 minutes served as the positive control. The rationale for using positive control was to show that ELISA kit is capable of determining 8-isoprostane and cleaved caspase 3, and lower levels of these bioactive molecules are not due to defects in the kit.

### 2.5. Cleaved-Caspase 3 Immunostaining

Cochleae harvested from rats at 7th day post-administration were washed in PBS three times and then sectioned. Cochlear sections were incubated with 5% normal goat serum (Sigma Aldrich, St Louis, MO, USA), 3% Triton X-100 (Sigma Aldrich, St Louis, MO, USA) in PBS for 30 minutes at 25 °C. Samples were incubated with the primary antibody of anti-cleaved caspase-3 (Asp175) rabbit polyclonal antibody (Cell Signaling, Danvers, MA, USA), and incubated overnight at 4 °C. Next day, samples were washed three times with PBS and incubated with Alexa Fluor 568 secondary antibody for 90 minutes at room temperature. After washing, the samples were mounted with mounting medium containing 4′,6-diamidino-2-phenylindole (DAPI) (Vector laboratories, Burlingame, CA, USA) and viewed under a confocal microscope (Carl Zeiss Microimaging, LLC; Thornwood, NY, USA). The cochlear tissues treated with cisplatin (10 µM) (Sigma Aldrich, St Louis, MO, USA) for 30 minutes served as the positive control. ImageJ software was used for processing and analyzing the images. For quantification, red signal intensity was measured, and the background was subtracted. The size of region of interest (ROI) was the same for all images.

### 2.6. Cytokine Determination:

The quantitative levels of TNF-α, IL-1β, IL-6 and IL-12 in whole cochlear homogenates were determined by ELISA using commercially available kits as per manufacturer’s instructions (ThermoFisher Scientific, Waltham, MA, USA).

### 2.7. TUNEL Staining

The extent of apoptosis in the cochlear sections at 7th day post-administration was determined by TUNEL staining using commercially available kit as per manufacturer’s instructions (ThermoFisher Scientific, Waltham, MA, USA). Slices exposed to recombinant DNase I (Sigma Aldrich, St Louis, MO, USA) at 37 °C for 10 min, which induced DNA strand breaks prior to the labeling procedures, served as the positive control. Cell nuclei was stained with DAPI and results were expressed as percentage TUNEL positive cells.

### 2.8. Statistical Analysis

The statistical analysis of data was performed using the Student’s *t* test and ANOVA using SPSS software (New York, NY, USA). *p* values <0.05 were considered statistically significant.

## 3. Results

### 3.1. Transtympanic Administration of BM-MSCs do not Induce Oxidative Stress in Rat Cochlea

**Figure 1:**
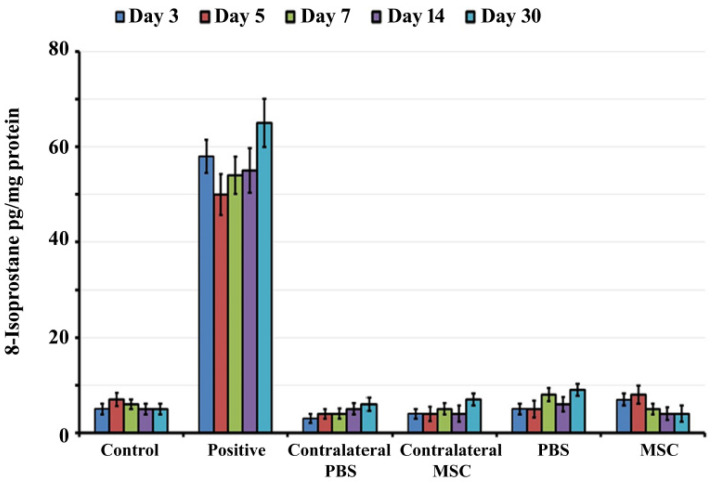
Oxidative stress determination: The levels of 8-isoprostane as a marker of oxidative stress was determined in whole cochlear tissue homogenates by ELISA at 3, 5, 7, 14, and 30 days post-administration. There was no statistically significant difference in 8-isoprostane levels in cochleae harvested from rats that received bone marrow mesenchymal stem cells (BM-MSCs), phosphate buffered saline (PBS) injected, or control group.

8-isoprostane is a well-accepted marker for oxidative stress in the cochlea [37,38]. Therefore, the levels of 8-isoprostane in whole cochlear tissue homogenates were determined by ELISA at 3, 5, 7, 14 and 30 day post-administration (Figure 1). There was no statistically significant difference in levels of 8-isoprostane between BM-MSCs treated, PBS injected, control and contralateral groups at all time periods (*p* > 0.05).

### 3.2. Caspase 3 Pathway is not Activated in Rat Cochlea in Response to BM-MSC Administration

**Figure 2:**
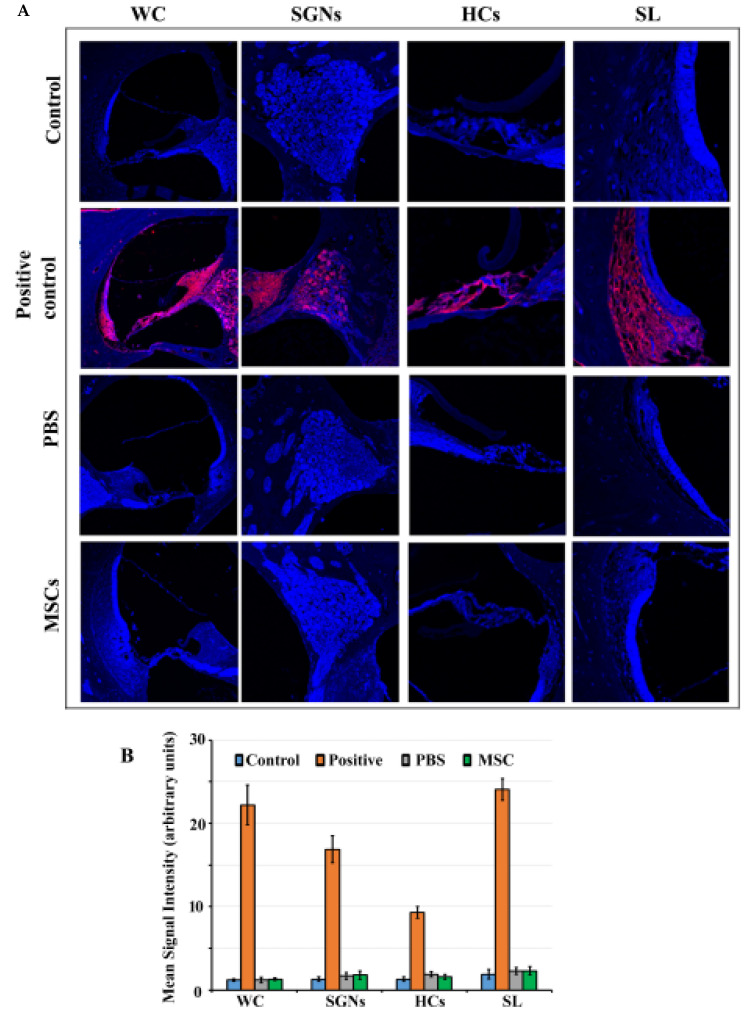
Cleaved caspase 3 immunostaining: (**A**) Rat cochlear slices were subjected to cleaved caspase 3 immunostaining (red) to determine apoptosis. Cell nuclei were stained with DAPI (blue). Cochleae harvested from rats that received BM-MSCs, PBS injected, or control group showed no or sparse staining whereas those from the positive group showed intense staining (red color). Blue color shows DAPI staining. (**B**) Mean signal intensity for cleaved caspase 3 was calculated using Image J software. Data are expressed as mean values ± standard deviation (SD). WC: whole cochlea; SGNs: Spiral Ganglion Neurons; HCs: Hair Cells; SL: Spiral Ligament.

Trans-tympanic administration of BM-MSCs and PBS do not induce the activation of the caspase 3 pathway as indicated by the absence of activated (cleaved) caspase 3 staining similar to control group (Figure 2A). There was no cleaved caspase 3 staining observable in the spiral ganglion neurons, organ of Corti and spiral ligament in control, PBS injected and BM-MSCs treated groups at 7th day post-administration. On the other hand, abundant cleaved caspase 3 staining was demonstrable in cisplatin treated cochlear slices (positive control). There was no statistically significant difference in mean signal intensity of cleaved caspase 3 staining between BM-MSCs treated, PBS injected and control groups (*p* > 0.05) (Figure 2B).

### 3.3. BM-MSCs did not Trigger Proinflammatory Cytokine Production in rat Cochlea

**Figure 3:**
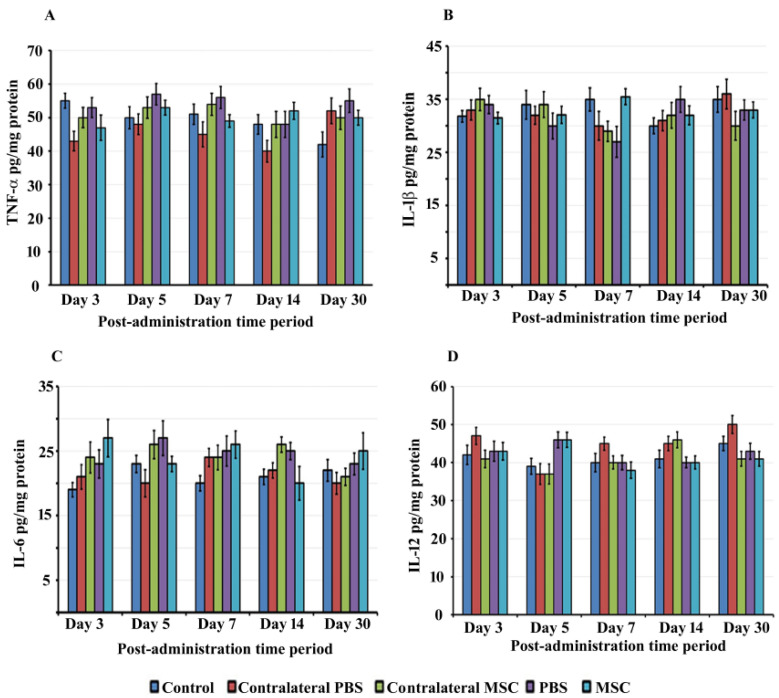
Proinflammatory cytokines: The levels of proinflammatory cytokines, tumor necrosis factor (TNF)-α (**A**), interleukin (IL)-1β (**B**), IL-6 (**C**) and IL-12 (**D**) were determined in cochlear homogenates by ELISA. Data are expressed as mean values ± SD.

The administration of foreign substances can trigger inflammatory responses in the cochlea that can cause auditory hair cell damage leading to hearing dysfunction. Therefore, we determined whether BM-MSCs induce the production of proinflammatory cytokines in the inner ear at different days post-administration. We did not observe the generation of TNF-α, IL-1β, IL-6 and IL-12 in the rat cochlea following administration of BM-MSCs determined by ELISA. There was insignificant difference in the levels of proinflammatory cytokines between BM-MSCs treated, PBS injected, control and contralateral groups at all time periods (*p* > 0.05) (Figure 3A–D).

### 3.4. BM-MSCs did not Induce Cell Death in Rat Cochlea

**Figure 4:**
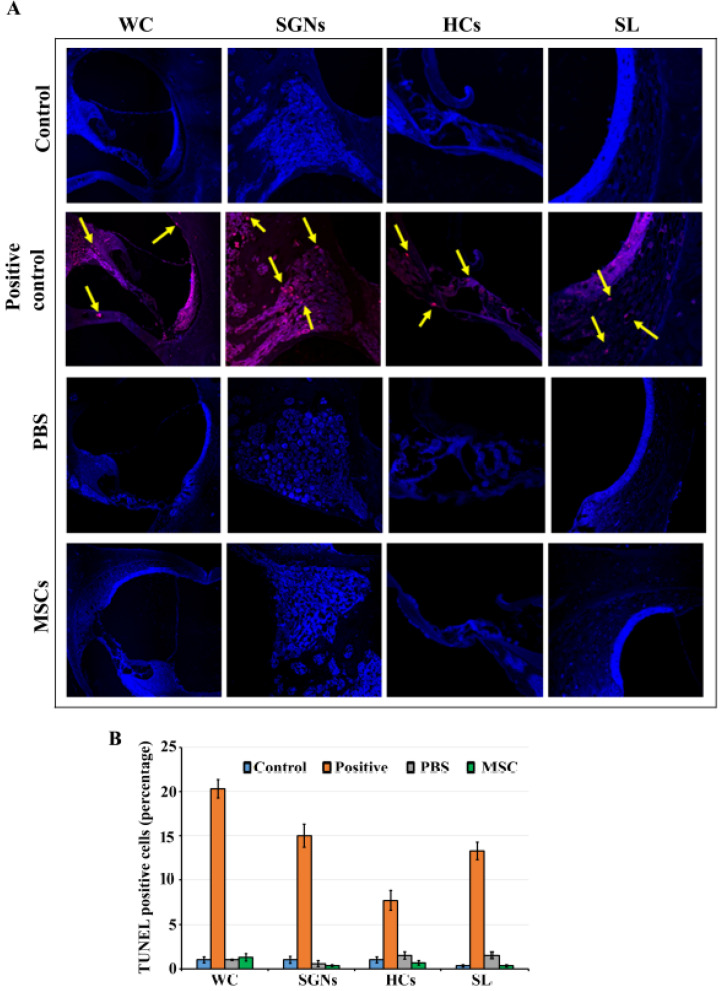
Transferase dUTP nick end labeling (TUNEL) staining: Rat cochlear slices were subjected to TUNEL immunostaining to determine cell death. (**A**) Cochleae harvested from rats that received BM-MSCs, PBS injected, or control group showed no or sparse staining whereas those from positive group showed intense staining (red color). Blue color shows DAPI staining. (**B**) The percentage of TUNEL positive cells were calculated and graphed. Arrows indicate cell death in positive control group. WC: whole cochlea; SGNs: Spiral Ganglion Neurons; HCs: Hair Cells; SL: Spiral Ligament.

Apoptosis in the rat cochlea in response to trans-tympanic administration of BM-MSCs was determined by TUNEL staining at 7th day post-administration using recombinant DNase I treated tissue slices as the positive control (Figure 4A). In the positive control group, abundant apoptotic cells were observed throughout the cochlea including cochlear hair cells, spiral ligament fibrocytes, the osseous spiral limbus, pericytes of the cochlea capillaries, Reissner’s membrane epithelial cells, the spiral ganglion satellite cells, endothelial cells, and stria vascularis (Figure 4A). However, we did not observe any apoptotic cells in the cochlea as indicated by the absence of red staining in the rat cochlea that received trans-tympanic administration of BM-MSCs or PBS similar to control group (Figure 4A). There was insignificant difference in the number of TUNEL positive cells between BM-MSCs treated, PBS injected, control and contralateral groups (Figure 4B).

## 4. Discussion

MSCs are a heterogeneous population of cells with the ability to differentiate into multilineage cell types [25,26,27] The ability to replace different tissue types coupled with their immunomodulatory properties have suggested the critical role that MSCs can play in tissue repair. One of the major mechanisms for tissue damage is oxidative stress [39]. Reactive oxygen species (ROS) are oxygen-containing molecules missing one electron that are highly reactive towards proteins, lipids, DNA, and RNA [40]. The oxidative stress results are due to the unpaired electron of the ROS destroying stable molecules [39,40]. MSCs are enriched in glutathione, an antioxidant, and are resistant to oxidative stress-induced cell death [41,42]. In addition, studies have shown the protective effect of MSCs on ulcer formation through regulating oxidative stress [43] and the ability of MSCs to rescue liver failure induced by carbon tetrachloride, a compound that induces lipid peroxidation and oxidative damage [44]. The antioxidant properties of MSCs, therefore, would be ideal to treat diseases where oxidative stress has been demonstrated to cause tissue damage.

ROS play a pivotal role in the ototoxicity inflicted on hair cells and spiral ganglion neurons. Increased concentration of superoxide has been found in human perilymph collected from patients with sensorineural hearing loss [45]. It has been demonstrated that noise exposure, ototoxic drugs, and aging can lead to increased ROS generation [46,47,48,49,50,51,52,53]. On the molecular level, oxidative stress causes DNA damage and lipid peroxidation in the cochlea [54,55,56]. Targeted mutation of the gene for cellular glutathione peroxidase (Gpx1) increases susceptibility to noise-induced hearing loss in animal models [57]. In the present study, we used the well-accepted 8-isoprotene as the marker of oxidative stress in the cochlea. We observed that administration of BM-MSCs does not induce oxidative stress in the rat cochlea.

Besides oxidative stress, proinflammatory cytokines are upregulated in the cochlea after inner ear insult. The proinflammatory cytokine levels can be of prognostic value and have been correlated with cochlear dysfunction, apoptosis of auditory sensory cells and the onset of SNHL [58]. These proinflammatory cytokines are produced by immune cells such as macrophages, leukocytes, and dendritic cells as well as neurons in the CNS in acute phase reactions [59,60]. Several histopathology studies have shown inflammatory changes in the cochlea in response to noise-induced hearing loss [61]. Fibroblasts in the cochlear lateral wall were observed to produce proinflammatory cytokines, TNF-α, IL-1β, and IL-6, which were increased after noise exposure [62]. TNF-α is among the most studied cytokines and has been linked to neurotoxicity, ROS generation, and neutrophil migration into the cochlea [63,64,65,66,67]. TNF-α was shown to cause a loss of function in the cochlea in vivo [68]. In the current study, we did not observe any difference in the levels of proinflammatory cytokine production in cochleae harvested from BM-MSCs treated, PBS injected, and control group. These findings suggest that BM-MSCs do not exert any inflammatory responses in the cochlea.

Administration of foreign substances into cochlea can lead to sensory cell damage leading to hearing impairment through induction of the apoptotic pathway [68]. Auditory hair cells undergo specific morphological changes during apoptosis, including cell membrane blebbing, cell body shrinkage, mitochondrial swelling, lost stereocilia, shrunken cuticular plates, disturbed junctional complexes, condensation of chromatin, nuclear fragmentation, and chromosome cleavage [69]. Apoptosis can occur via the intrinsic pathway through mitochondrial damage, or the extrinsic pathway via death receptor pathways [69]. In both pathways, caspases play important roles in the signaling cascade [69,70]. In the cochlea, the caspase 3 pathway is one of the major pathways that triggers apoptosis [69,70]. Caspase 3 is a member of the caspase family of cysteine proteases in the apoptosis pathway [71,72,73]. Under normal physiological conditions, caspase 3 exists as an inactive procaspase. Upon damage, the pro-domain is cleaved and caspase 3 is activated to cleave a specific tetrapeptide sequence of intracellular target proteins [61]. In the intrinsic pathway, activated caspase 3 ultimately activates caspase-activated DNase (CAD), which fragments cell DNA leading to cell death [69,70,74]. The activated caspase 3 also initiates the activation of apoptotic chromatin condensation inducer in the nucleus (ACINUS) via cleavage of cytosolic helicase with an *N*-terminal caspase-recruitment domain (HELI-CARD) [75], causing DNA fragmentation. In the extrinsic pathway, initiator caspases are activated upon binding and dimerization of a ligand to a death receptor [69]. This event forms a death-inducing signaling complex (DISC) that then cleaves and promotes effector caspases such as activated caspase 3, leading to cell death [69].

Caspase 3 has a major role in apoptosis in hair cells and is responsible for hair cell damage after ototoxic insult to the inner ear [76]. The most common agents are cisplatin and aminoglycoside antibiotics [77]. The outer hair cells of the basal turn of the cochlea are especially sensitive to aminoglycosides [77]. Injury to the auditory hair cells after drug toxicity leads to expression of pro-inflammatory factors and free radicals, causing oxidative stress [68]. Oxidative stress induces the intrinsic apoptotic pathway via Bax activation, caspase 3, and mitochondrial and DNA damage [69]. Cisplatin, in addition to injuring outer hair cells of the basal turn, also injures spiral ganglion neurons, the first order neurons in the central auditory pathway via release of oxidative free radicals [78]. The role of caspase 3 in drug-induced ototoxicity to the inner ear has been shown with pan-caspase inhibitors [69]. It was shown that inhibition of caspases with pan-caspase inhibitors prevents the loss of Corti cells and spiral ganglion neurons exposed to gentamicin and cisplatin [71,72]. These studies clearly demonstrate that caspase 3 can be used as a biomarker of sensory cell damage in the cochlea. In this study, we observed that trans-tympanic administration of BM-MSCs does not induce the activation of the caspase 3 pathway as indicated by the absence of cleaved caspase 3 staining in cochlear sections. On the other hand, a strong cleaved caspase 3 staining was evident in cochlear sections treated with cisplatin that served as positive control.

Terminal deoxynucleotidyl transferase (TdT) dUTP Nick-End Labeling (TUNEL) assays are used to detect cells in the late stages of apoptosis, after they have undergone DNA fragmentation and degradation by endonucleases [79,80,81]. This produces DNA fragments with double-stranded breaks and produces the characteristic “laddering” pattern visualized by gel electrophoresis. Exogenous TdT labels the 3′-OH ends of double-stranded DNA strand breaks with fluorochromes, without the need for a template. Apoptotic cells are then identified using immunohistochemical techniques such as flow cytometry or fluorescence microscopy [77]. In this study, we observed that there was no impact of trans-tympanic administration on the initiation of cell death pathway in the cochlea.

In the present study, we used trans-tympanic administration of BM-MSCs. However, previous studies used direct injection of MSCs into the inner ear. One early study found that after direct injection into the lateral semicircular canal, MSCs were detected at the site of injury and auditory brainstem response (ABR) thresholds at 40 kHz improved by 23% in rats that had lateral wall invasion by MSCs [81]. However, the administration and migration of stem cells throughout the cochlea is not a simple process. Different sites of administration yield varying results and are associated with different risks. Administration to the Rosenthal’s canal and peri-lymphatic spaces show early promising results; however, cells do not survive well in endolymphatic administration, likely due to the high concentrations of potassium (K+) in endolymph [82]. Transportation into the modiolus and the cochlear nerve show better results but also carries a higher risk of inadvertent structural damage [83]. Overall, the benefits of direct injection of stem cells to the cochlea is associated with challenges that need to be addressed. In addition to the cell’s uncertain functionality, direct cochlear administration is complicated by the invasiveness of the procedure, may require immunosuppression, and there is a prospect of cochlear damage during the procedure, leading to further hearing impairment. Trans-tympanic injection limits these drawbacks while providing comparable benefits.

The limitation of the present study is that we have not studied the distribution of BM-MSCs into the Eustachian tube which needs to be determined in future studies. Another limitation of our study is lack of assessment as to whether MSCs actually entered the inner ear. Future studies employing fluorescent conjugated BM-MSCs will help in addressing this concern. However, it has been demonstrated that BM-MSCs can exert protective effects through their paracrine mechanisms [17,84,85,86,87]. While previously it was thought that MSCs directly and actively participated in the tissue repair and regeneration process, there is increasing evidence that MSCs do not engraft within tissue [88]. It has been demonstrated that the majority of tissue protective effects of MSCs are exerted via paracrine signaling mechanisms. Increasing evidence shows that MSCs secrete extracellular vesicles containing a diverse array of bioactive factors collectively called the secretome [89,90,91,92,93,94]. These include angiogenic and anti-inflammatory molecules such as IGF-1, VEGF-D, and IL-10, as well as transcriptional regulators microRNA, transfer RNA, long noncoding RNA, growth factors, proteins, and lipids [17,94]. Since all these biologically active factors have small molecular weight, it is reasonable to speculate that they can pass from middle to inner ear and can exert their otoprotective effects. Indeed, intraperitoneal injection of human adipose tissue derived MSCs (hASCs) led to restoration of hearing and protection of auditory hair cells in a mouse model of autoimmune hearing loss [94]. Regeneration of spiral ganglion cells was observed, which were of mouse origin and not from human stem cells that were infused into the mice. It was concluded that hASCs provided oto-protection through their paracrine molecular mechanisms via secretion of biologically active molecules. Additionally, MSCs can also reduce ROS-mediated tissue damage by upregulating anti-oxidative enzymes, as well as transfer mitochondria in damaged cells [94]. This combination of functions is particularly useful as it allows MSCs to reduce existing free radicals via antioxidant enzymes, eliminate the source of new free radicals by replacing dysfunctional mitochondria, and start the process of tissue repair by stimulating angiogenesis. In addition, studies have shown benefits of systemic and subarachnoid administration of stem cells on hearing loss and detected injected stem cells in the inner ear [95,96]. Umbilical cord blood derived MSCs (UCB-MSCs) were observed near spiral ganglion neurons and sensory hair cells from apical to basal cochlear turns when injected into the brachial vein in a guinea pig model of sensorineural hearing loss [95]. On par with these findings, umbilical cord derived MSCs (UMSCs) were demonstrable in different regions of the cochlea, including the stria vascularis, the basal membrane and the spiral ganglions following administration into the subarachnoid cavity in congenital deaf albino pigs [96]. Moreover, the round window membrane (RWM) may not be as strictly regulated as the blood brain barrier (BBB). Recent reviews indicate that the RWM behaves as if it is semi-permeable and even has microvilli on its apical surface which speaks to its absorptive properties [97]. These findings suggest that MSCs have the ability to penetrate tight barriers such blood labyrinth barriers (BLB), and potentially the round window membrane (RWM), to reach the cochlear sensory compartment.

In summary, the results of the present study suggest that the main critical inflammatory mechanisms and apoptotic pathways were not activated by the trans-tympanic administration of MSCs. Trans-tympanic injection is a feasible approach for non-surgical administration of compounds from middle to inner ear as it is non-invasive and will not have any adverse effects on sensory structures that may have implications in hearing impairment. Trans-tympanic administration of BM-MSCs does not result in oxidative stress or inflammatory response in the immunocompetent rat cochlea. In future studies, it will be worthwhile to explore the potential of BM-MSCs in providing oto-protection for auditory disorders such as noise trauma and ototoxicity.
